# Cross-platform analytical assessment of serum GFAP quantification in multiple sclerosis: SIMOA versus two automated immunoassays

**DOI:** 10.3389/fneur.2025.1682198

**Published:** 2025-10-20

**Authors:** Jordi Tortosa-Carreres, Laura Cubas-Núñez, Jéssica Castillo-Villalba, Lorena Forés-Toribio, Raquel Gasque-Rubio, Carlos Quintanilla-Bordas, Carmen Alcalà-Vicente, Sara Carratalà-Boscà, Ana Vaño-Bellver, Begoña Laiz-Marro, Francisco Carlos Pérez-Miralles, Bonaventura Casanova

**Affiliations:** ^1^Laboratory Department, La Fe University and Polytechnic Hospital, Valencia, Spain; ^2^Neuroimmunology Research Group, Health Research Institute La Fe, Valencia, Spain; ^3^Neurology Department, General University Hospital of Castellón, Castelló de la Plana, Spain; ^4^Neurology Department, University Hospital of La Ribera, Valencia, Spain; ^5^Neuroimmunology Unit, La Fe University and Polytechnic Hospital, Valencia, Spain

**Keywords:** sGFAP, multiple sclerosis, biomarkers, SIMOA, Lumipulse, Alinity

## Abstract

**Introduction:**

Serum glial fibrillary acidic protein (sGFAP) is a promising biomarker, but its quantification mainly relies on SIMOA, a technology not widely available in clinical practice.

**Objectives:**

To evaluate the analytical performance of two high-throughput automated platforms—Alinity^®^ i (Abbott) and Lumipulse^®^ G1200 (Fujirebio)—for sGFAP quantification.

**Methods:**

A retrospective longitudinal study included 107 serum samples from 23 MS patients. sGFAP was measured with SIMOA SR-X^®^, Lumipulse^®^ G1200, and Alinity^®^ i. Data were log-transformed. Agreement was assessed using Pearson correlations, Passing–Bablok regression, Bland–Altman analysis, and Δlog correlations between visits. Longitudinal differences across platforms were tested with a linear mixed-effects model (platform as fixed effect, SIMOA as reference). Moreover, ΔSIMOA was modeled against ΔLumipulse and ΔAlinity, adjusting for ΔEDSS, phenotype, relapses and new MRI lesions.

**Results:**

Passing–Bablok regression yielded slopes of 0.85 (SIMOA–Lumipulse), 0.81 (SIMOA–Alinity), and 0.95 (Lumipulse–Alinity), with intercepts of −0.32, −0.35, and −0.05. Mean log-biases were −0.622, −0.733, and 0.109. Correlations between log-means and log-differences were *r* = 0.26 (*p* = 0.006), 0.44 (*p* < 0.0001), and 0.15 (*p* = 0.13). The mixed-effects model showed no significant Δlog differences relative to SIMOA (*p* > 0.1). When modeling ΔSIMOA, ΔLumipulse was a significant predictor (*β* = 0.51; *p* = 0.002), whereas ΔAlinity showed only a trend (*β* = 0.31; *p* = 0.051). No clinical covariates were significantly associated.

**Conclusion:**

Automated platforms, particularly Lumipulse, showed strong concordance with SIMOA supporting the role in analytical monitoring.

## Introduction

Serum biomarkers are transforming neurology by offering minimally invasive tools for diagnosis, prognosis, and disease monitoring. Among these, serum glial fibrillary acidic protein (sGFAP)—a 50-kDa astrocytic filament protein—has emerged as a promising marker of reactive astrogliosis and astrocytic injury (1–3).

Elevated sGFAP has been linked to progressive multiple sclerosis (MS), where it correlates with greater disease burden and aggressive phenotypes (4–6), as well as to amyloid/tau pathology and cognitive decline in Alzheimer’s disease (7–10). Increased levels have also been reported in Neuromyelitis Optica Spectrum Disorder (NMOSD), reflecting relapse severity (11, 12), and in amyotrophic lateral sclerosis, particularly with coexisting Alzheimer’s pathology (13).

Quantification of sGFAP is most frequently performed using the ultrasensitive SIMOA® (Single Molecule Array) platform (Quanterix Corporation, Massachusetts, United States), a digital ELISA-based technology. However, its technical complexity and operational demands limit its implementation to specialized laboratories and tertiary care centers (14–16).

More recently, fully automated systems such as Lumipulse® (Fujirebio Inc., Tokyo, Japan) and Alinity® i (Abbott Laboratories, Illinois, United States) have introduced sGFAP assays designed for integration into high-throughput clinical workflows. The Lumipulse platform has already demonstrated strong concordance with SIMOA for other neurological biomarkers, including pTau181 (17) and total Tau, Aβ42 and Aβ40 (18, 19), as well as neurofilament light chain (20). In parallel, the Alinity platform has been incorporated into diagnostic algorithms for traumatic brain injury, in combination with Ubiquitin C-terminal Hydrolase L1, to support rapid decision-making in emergency care settings (21–23). Although preliminary reference ranges for Lumipulse have been reported (24), no head-to-head comparisons across automated platforms and SIMOA have yet been conducted in MS.

The present study aimed to directly compare sGFAP concentrations measured by SIMOA, Lumipulse, and Alinity in patients with MS, in order to evaluate their analytical agreement and clinical interoperability.

## Materials and methods

A retrospective study was conducted using longitudinal serum samples from patients with clinically confirmed MS, recruited at the Department of Neurology, Hospital Universitari i Politècnic La Fe (Valencia, Spain). Patients were followed prospectively for 2 years, with serum collected every 4 months, yielding 105 samples from 23 individuals. Inclusion criteria were: (i) MS diagnosis according to the 2017 revised McDonald criteria (25), established by three experienced neurologists specialized in MS; (ii) systematic exclusion of alternative motor neuron and other neurological disorders through detailed clinical assessment and complementary investigations; (iii) signed informed consent; and (iv) availability of at least two longitudinal serum samples.

Clinical variables included age, sex, disease duration, disease-modifying therapy (DMT), Expanded Disability Status Scale (EDSS), relapses, new magnetic resonance imaging (MRI) lesions, and EDSS worsening defined by harmonized criteria for both magnitude and temporal confirmation of clinically meaningful and sustained progression (26). Relapses and new MRI lesions were assigned to the serum sampling time point closest to the clinical event or lesion detection, respectively.

### Sample collection

Serum samples were obtained by venipuncture into gel-separator tubes, centrifuged at 2000 g for 10 min after 30 min at room temperature, aliquoted, stored at 4 °C for <24 h, and frozen at −80 °C until analysis. All samples were provided by the Neuroimmunology Research Group, Hospital Universitari i Politècnic La Fe (Valencia, Spain).

### Laboratory analysis

On the SIMOA platform, the Neurology 2-Plex B kit (Ref#: 103520) was used with the SIMOA SR-X® system. This method employs a digital sandwich ELISA in which sGFAP present in the sample binds to paramagnetic beads coated with specific monoclonal antibodies, followed by a biotinylated detector antibody. After a wash step, streptavidin-*β*-galactosidase is introduced, binding to biotin and catalyzing the conversion of a fluorescent substrate (β-D-galactopyranoside). Beads are individually compartmentalized into thousands of femtoliter-sized microwells and scanned to detect single-molecule fluorescence signals, enabling ultra-sensitive quantification. The functional lower limit of quantification (LLOQ) was 16.6 pg./mL, with a validated dynamic range of 16.6–40,000 pg./mL.

In the case of Lumipulse, analyses were conducted on the Lumipulse® G1200 system using the Lumipulse® G GFAP assay (Ref#: 261255). This is a two-step chemiluminescent sandwich immunoassay. Initially, sGFAP in the sample binds to microparticles coated with murine monoclonal anti-GFAP antibodies. After washing, a second murine monoclonal antibody conjugated to alkaline phosphatase (ALP) is added. The resulting chemiluminescent signal, produced through ALP-mediated hydrolysis of AMPPD (3-(2′-spiroadamantane)-4-methoxy-4-(3″-phosphoryloxy) phenyl-1,2-dioxetane disodium salt), is directly proportional to sGFAP concentration. The LLOQ was 16.6 pg./mL, and the analytical range was 10–5,000 pg./mL.

In the case of Alinity, sGFAP was quantified on the Alinity i system (Abbott, Illinois, United States) with the GFAP Reagent Kit (Ref#: 04 W1720). This assay utilizes a chemiluminescent microparticle immunoassay format. In the first incubation step, sGFAP in the sample binds to paramagnetic microparticles coated with murine monoclonal anti-GFAP antibodies. After washing, an acridinium-labeled murine monoclonal antibody is added. Following a second wash, chemiluminescence is triggered via chemical activation of the acridinium label and measured in relative light units (RLU) by the system’s optical detector. The LLOQ was 3.2 pg./mL, with a validated analytical range of 6.1–42,000 pg./mL. The manufacturer-recommended reference value is <35 pg./mL for the TBI detection.

All procedures followed manufacturer instructions. Quality control was ensured using internal controls (two levels for SIMOA and Lumipulse, three for Alinity). Calibration yielded expected values, with all control and patient samples within range. A single reagent lot was used for each platform, with two replicates of each control level per run, and patient samples analyzed in duplicate runs.

Specifically, for SIMOA, intra-assay CVs were 14.54% at level 1 and 7.41% at level 2, with inter-assay CVs of 6.1 and 10.2%, respectively. For Lumipulse, intra-assay CVs were 4.42% at level 1 and 0.07% at level 2, with inter-assay CVs of 2.15 and 1.58%. For Alinity, intra-assay CVs were 4.34, 1.24, and 2.49%, while inter-assay CVs were 2.86, 4.68, and 3.55% across the different control levels.

### Statistical analysis

All statistical analyses were performed using RStudio (version 4.3.2). sGFAP values were log-transformed to normalize measurement scales across platforms, given that SIMOA concentrations predominantly fell within the hundreds range, whereas Alinity and Lumipulse measurements were generally in the tens. Normality of continuous variables was assessed using the Shapiro–Wilk test.

Interchangeability between the three analytical platforms was assessed using correlation analysis, Passing–Bablok regression, and Bland–Altman methodology. The *mcr* package was used to compute Passing–Bablok regression parameters and generate the corresponding plots. Bland–Altman estimators were obtained with the *BlandAltmanLeh* package, and visualizations were generated using custom scripts based on *ggplot2*. To evaluate proportional bias, the correlation between measurement differences and their corresponding means was examined within the Bland–Altman framework.

To assess longitudinal concordance of sGFAP dynamics across platforms, relative changes between two consecutive measurements were calculated as logarithmic differences (Δlog), defined as:


$$\Delta \log_{i}^{\left(t\right)}=\log\;(x_{i}^{\left(t\right)})-\log\;(x_{i}^{\left(t-1\right)})$$

Where $\Delta \log_{i}^{\left(t\right)}$ represents the logarithmic change for platform *i* at time t, and $x_{i}^{\left(t\right)}$ denotes the observed sGFAP concentration at that time point.

Correlations between Δlog values were estimated to assess the directional consistency of changes across platforms. To investigate potential systematic differences in the magnitude of change, we fitted a linear mixed-effects model to the Δlog values with platform (SIMOA, Lumipulse, Alinity) as a fixed effect—using SIMOA as the reference—and a patient-specific random intercept to account for repeated measures; models were fitted with *lme4*.

In a complementary analysis, we assessed whether changes detected by SIMOA could be predicted from those captured by the other platforms while accounting for clinical activity. Mixed-effects regressions were fitted with ΔSIMOA as the dependent variable and ΔLumipulse and ΔAlinity as main predictors, adjusting for ΔEDSS ( ${\text{EDSS}}_{t}-\mathrm{EDS}S_{t-1}$ ), clinical phenotype, relapses, and new lesions, and including a patient-specific random intercept. Models were fitted with *lme4* and *lmerTest*. To focus on analytical agreement, no additional covariates (age, sex, treatment) were included to avoid unnecessary overadjustment. *p* values for the two primary predictors (ΔLumipulse, ΔAlinity) were Holm-adjusted, whereas exploratory covariates were evaluated under false discovery rate (FDR, Benjamini–Hochberg) control.

In both models, patient-specific random intercepts were modeled as normally distributed with mean zero and estimated variance to account for repeated measures, as implemented in the *lme4* package.

Associations between sGFAP values and EDSS across all determinations were evaluated using correlation coefficients. Within-patient changes were examined by correlating Δlog values from each platform with the corresponding ΔEDSS. Comparisons between patients with and without relapse or new MRI lesions were performed using t-tests. Correlations of baseline sGFAP with age and disease duration were also assessed, and baseline levels were compared between phenotypes (relapsing–remitting vs. progressive [SP + PP]) using t-tests.

Pearson correlation coefficients were used for all continuous variables. Spearman coefficients were applied exclusively for correlations with EDSS, given its ordinal scale. Statistical significance was set at *p* < 0.05. For Passing–Bablok and Bland–Altman analyses, significance was defined as 95% confidence intervals excluding the null (0 for intercepts and mean differences; 1 for slopes).

Data wrangling was performed with the *dplyr* and *tidyr* packages, and visualizations were generated using *ggplot2* and *ggsignif*.

### Ethical statement

The methodology of the current research was approved by the Institutional Ethics Committee (reference number PI20/01446). The study procedures adhered to the principles of the Declaration of Helsinki, and all necessary measures were taken to ensure data confidentiality.

## Results

A total of 105 serum samples from 23 patients diagnosed with MS were analyzed. For the Alinity platform, 9 samples were not processed due to insufficient volume. Table 1 summarizes the baseline demographic and clinical characteristics of the patients. Across all determinations, median raw sGFAP concentrations were 123.68 pg./mL (IQR: 88.74) for SIMOA, 28.40 pg./mL (IQR: 15.80) for Lumipulse, and 21.90 pg./mL (IQR: 10.97) for Alinity. Corresponding log-transformed values yielded means of 2.08 ± 0.21, 1.46 ± 0.18, and 1.34 ± 0.17, respectively.

**Table 1:** Demographic and clinical characteristics, as well as raw and log-transformed sGFAP values, of patients with multiple sclerosis at baseline.

	RRMS (*n* = 9)	SPMS (*n* = 8)	PPMS (*n* = 6)	Total (*n* = 23)
Sex (M/F)	0/9	4/4	1/5	5/18
Age, years (mean ± SD)	38.5 ± 7.19	46.7 ± 2.69	54.2 ± 7.19	45 ± 10
Disease duration, (mean ± SD)	6.5 ± 6.52	8.6 ± 7.23	18.5 ± 6	11.4 ± 8.42
DMT (*n*, %)				
Glatiramer Acetate	1 (11.1%)	0 (0%)	0 (0%)	1 (4.4%)
Rituximab	1 (11.1%)	3 (37.5%)	3 (50%)	7 (30.4%)
Teriflunomide	5 (55.6%)	0 (0%)	0 (0%)	5 (21.7%)
Fingolimod	0 (0%)	2 (25%)	0 (0%)	2 (8.7%)
Ocrelizumab	1 (11.1%)	1 (12.5%)	1 (16.7%)	3 (13.1%)
No DMT	1 (11.1%)	2 (25%)	2 (33.3%)	5 (21.7%)
EDSS, median (IQR)	1 (1)	3.5 (4)	5 (2)	3 (4)
sGFAP Simoa, median (IQR)	133 (76.3)	143.9 (86.8)	182.3 (63.7)	144.7 (85.9)
sGFAP Lumipulse, median (IQR)	27.8 (15.6)	35.8 (17.3)	37.6 (12)	33.4 (16.6)
sGFAP Alinity, median (IQR)	20.2 (10.7)	28.7 (15.5)	26.7 (12.5)	22.8 (14.4)
Log (sGFAP) Simoa, (mean ± SD)	2 ± 0.2	2.16 ± 0.17	2.21 ± 0.15	2.12 ± 0.21
Log (sGFAP) Lumipulse, (mean ± SD)	1.38 ± 0.22	1.51 ± 0.15	1.55 ± 0.11	1.46 ± 0.18
Log (sGFAP) Alinity, (mean ± SD)	1.25 ± 0.18	1.36 ± 0.17	1.39 ± 0.15	1.32 ± 0.18

Continuous variables with normal distribution are presented as mean ± standard deviation (SD); non-normally distributed and ordinal variables as median (interquartile range, IQR); and categorical variables as number (percentage, %). Baseline age and disease duration were normally distributed (Shapiro–Wilk test, *p* ≈ 0.15). M, Male; F, Female; SD, Standard Deviation; MS, Multiple sclerosis; PPMS, Primary progressive MS; RRMS, Relapsing–remitting MS; SPMS, Secondary progressive MS; DMT, Disease-modifying therapy; EDSS, Expanded Disability Status Scale; IQR, Interquartile Range; Gd, Gadolinium; sGFAP, serum Glial fibrillary acidic protein.

The cohort comprised 5 males (22%) and 18 females (78%), with a mean baseline age of 45 ± 11 years. Clinical phenotypes included 9 relapsing–remitting (39%), 8 secondary progressive (35%), and 6 primary progressive (26%) patients, with no conversions from relapsing–remitting to secondary progressive during follow-up. At baseline, three patients had lesion accrual within the preceding 90 days, although all had been relapse-free for >90 days. During follow-up, four patients developed new lesions and two experienced relapses, with GFAP measurements obtained 23 and 56 days after the events. Thirteen minor EDSS increases and 18 minor decreases were observed, none of which met the criteria for clinically meaningful or sustained change.

Supplementary Figures S1–S3 display data distribution of raw (non–log-transformed) and log-transformed sGFAP concentrations across the three platforms, together with Shapiro–Wilk test results.

### Passing–Bablok regression

**Figure 1:**
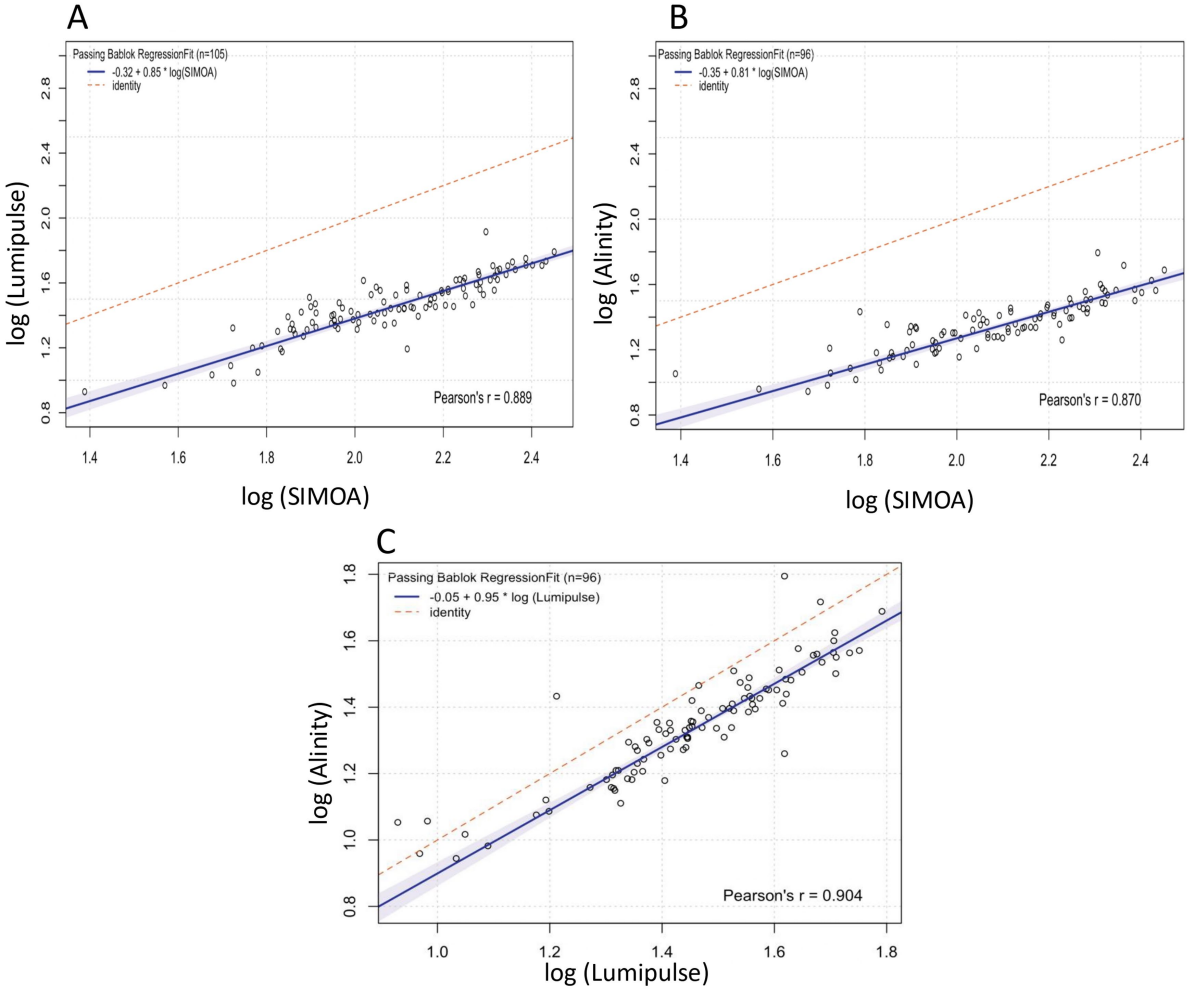
Passing–Bablok regression analysis comparing quantitative agreement across the three analytical platforms. Panels **(A–C)** correspond to the SIMOA–Lumipulse, SIMOA–Alinity, and Lumipulse–Alinity comparisons, respectively.

Passing–Bablok regression between SIMOA and Lumipulse showed an intercept of −0.32 log (95% CI: −0.47, −0.15) and a slope of 0.85 log (95% CI: 0.77, 0.92), with a Pearson correlation of *r* = 0.89 (*p* < 0.0001; Figure 1A). For the SIMOA–Alinity pair, the intercept was −0.35 log (95% CI: −0.49, −0.18) and the slope 0.81 log (95% CI: 0.72, 0.88), with a Pearson correlation of *r* = 0.87 (*p* < 0.0001; Figure 1B). The Lumipulse–Alinity regression yielded an intercept of −0.05 log (95% CI: −0.27, 0.04) and a slope of 0.95 log (95% CI: 0.88, 1.03), with a Pearson correlation of *r* = 0.90 (*p* < 0.0001; Figure 1C).

### Bland–Altman analysis

**Figure 2:**
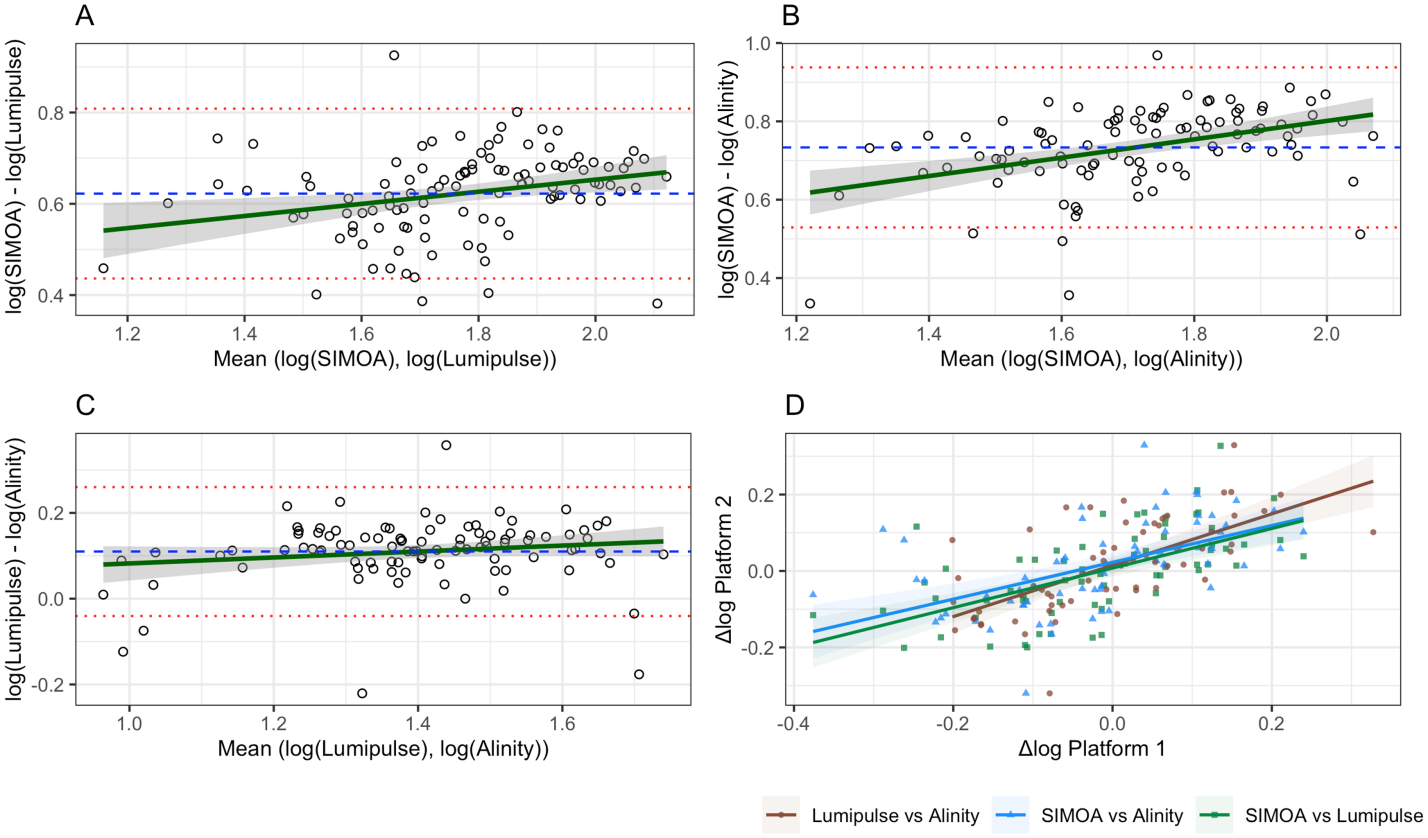
Bland–Altman plots and correlation analysis of log-transformed variability (Δlog) between platforms. Panels **(A–C)** represent Bland–Altman analyses: the dashed blue line indicates the mean bias, red lines represent the upper and lower limits of agreement, and the green line depicts the regression line for the correlation between log-means and log-differences. Panel **(D)** shows Pearson correlations between Δlog values for each pair of platforms.

In the analysis of Lumipulse versus SIMOA, the mean bias was −0.62 log (95% CI: −0.64 to −0.60), with limits of agreement from −0.81 (95% CI: −0.84 to −0.78) to −0.44 (95% CI: −0.47 to −0.41). A significant correlation was observed between the differences and the means (*r* = 0.26; *p* = 0.006; Figure 2A).

For Alinity versus SIMOA, the mean bias was −0.73 log (95% CI: −0.76 to −0.71), with limits of agreement between −0.94 (95% CI: −0.97 to −0.90) and −0.53 (95% CI: −0.57 to −0.49). A stronger correlation was found (*r* = 0.44; *p* < 0.0001; Figure 2B).

In the Lumipulse–Alinity comparison, the mean bias was 0.11 log (95% CI: 0.02 to 0.19), with limits of agreement from −0.040 (95% CI: −0.21 to 0.13) to 0.26 (95% CI: 0.09 to 0.43). No significant correlation was detected (*r* = 0.15; *p* = 0.13; Figure 2C).

Table 2 summarizes all statistical estimates reported in Sections 3.1 and 3.2. Analogous versions of Figures 1, 2A–C, as well as a counterpart to Table 2 based on raw sGFAP values, are provided in Supplementary Table S1 and Supplementary Figures S4, S5, respectively.

**Table 2:** Pairwise method comparison and agreement between SIMOA, Lumipulse, and Alinity platforms.

	SIMOA vs Lumipulse	SIMOA vs Alinity	Lumipulse vs Alinity
Intercept (log) [95%CI]*	−0.32 [−0.47, −0.15]	−0.35 [−0.49, −0.18]	−0.05 [−0.27, 0.04]
Slope (log) [95%CI]*	0.85 [0.77, 0.92]	0.81 [0.72, 0.88]	0.95 [0.88, 1.03]
Log (sGFAP) Correlation (r)	0.89 (*p* < 0.0001)	0.87 (*p* < 0.0001)	0.90 (*p* < 0.0001)
Bias (log) [95%CI]†	−0.62 [−0.64, −0.60]	−0.73 [−0.76, −0.71]	0.109 [0.02, 0.19]
LL (log) [95%CI]†	−0.81 [−0.84, −0.78]	−0.94 [−0.97, −0.90]	−0.04 [−0.21, 0.13]
UL (log) [95%CI] †	−0.44 [−0.47, −0.41]	−0.53 [−0.57, −0.49]	0.26 [0.08, 0.43]
Bias-Mean Correlation (r)	0.26 (*p* = 0.006)	0.44 (*p* < 0.0001)	0.15 (*p* = 0.13)

*Passing–Bablok regression estimates; †Bland–Altman analysis estimates. LL, Lower Limit of Agreement; UL, Upper Limit of Agreement; CI: Confidence Interval; sGFAP, Serum Glial Fibrillary Acidic Protein; r, Pearson correlation coefficient.

### Longitudinal analysis

Correlations between Δlog values were statistically significant across platforms: SIMOA vs. Lumipulse, *r* = 0.60, *p* < 0.001; SIMOA vs. Alinity, *r* = 0.55, *p* < 0.001; Lumipulse vs. Alinity, *r* = 0.65, *p* < 0.001 (Figure 2D).

In the linear mixed-effects model comparing Δlog values across platforms, the intercept corresponding to SIMOA was *β* = −0.023 (SE = 0.01, *p* = 0.24). The estimated difference relative to SIMOA was *β* = 0.018 (SE = 0.02, *p* = 0.36) for Lumipulse and *β* = 0.033 (SE = 0.02, *p* = 0.12) for Alinity. Variability explained by the patient-specific random intercept was negligible (SD = 0.026; 95% CI: 0.0001, 0.027).

In a complementary mixed-effects model, ΔLumipulse emerged as a significant predictor of ΔSIMOA (*β* = 0.51, SE = 0.16, *p* = 0.002), whereas ΔAlinity showed only a trend toward significance (*β* = 0.31, SE = 0.15, *p* = 0.051). By contrast, ΔEDSS (*p* = 0.70), relapse occurrence (*p* = 0.90), new MRI lesions (*p* = 0.44), and clinical phenotype (all *p* > 0.85) were not associated with ΔSIMOA. After Holm adjustment for the two prespecified primary predictors, ΔLumipulse remained significant (P_adj_ = 0.004), while ΔAlinity remained non-significant (P_adj_ = 0.05).

**Figure 3:**
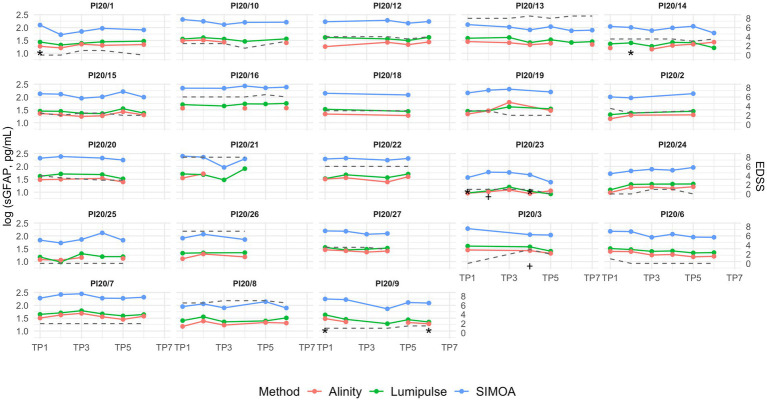
Longitudinal serum GFAP concentrations in patients with multiple sclerosis over a 2-year follow-up, with samples collected every 4 months. Missing data points indicate unavailable serum aliquots for the respective platform. The grey dashed line represents EDSS trajectory. TP = time point (TP1–TP7 correspond to sequential visits approximately at baseline, 4, 8, 12, 16, 20, and 24 months); sGFAP: serum GFAP. * indicates the time point at which the patient showed an increase in lesion burden during the interval since the previous visit; † indicates the time point at which the patient experienced a clinical relapse during the interval since the previous visit.

Figure 3 illustrates the longitudinal dynamics of serum GFAP levels across the three analytical platforms; corresponding analyses using raw values are provided in Supplementary Figure S6.

### Association with clinical and demographic variables

**Figure 4:**
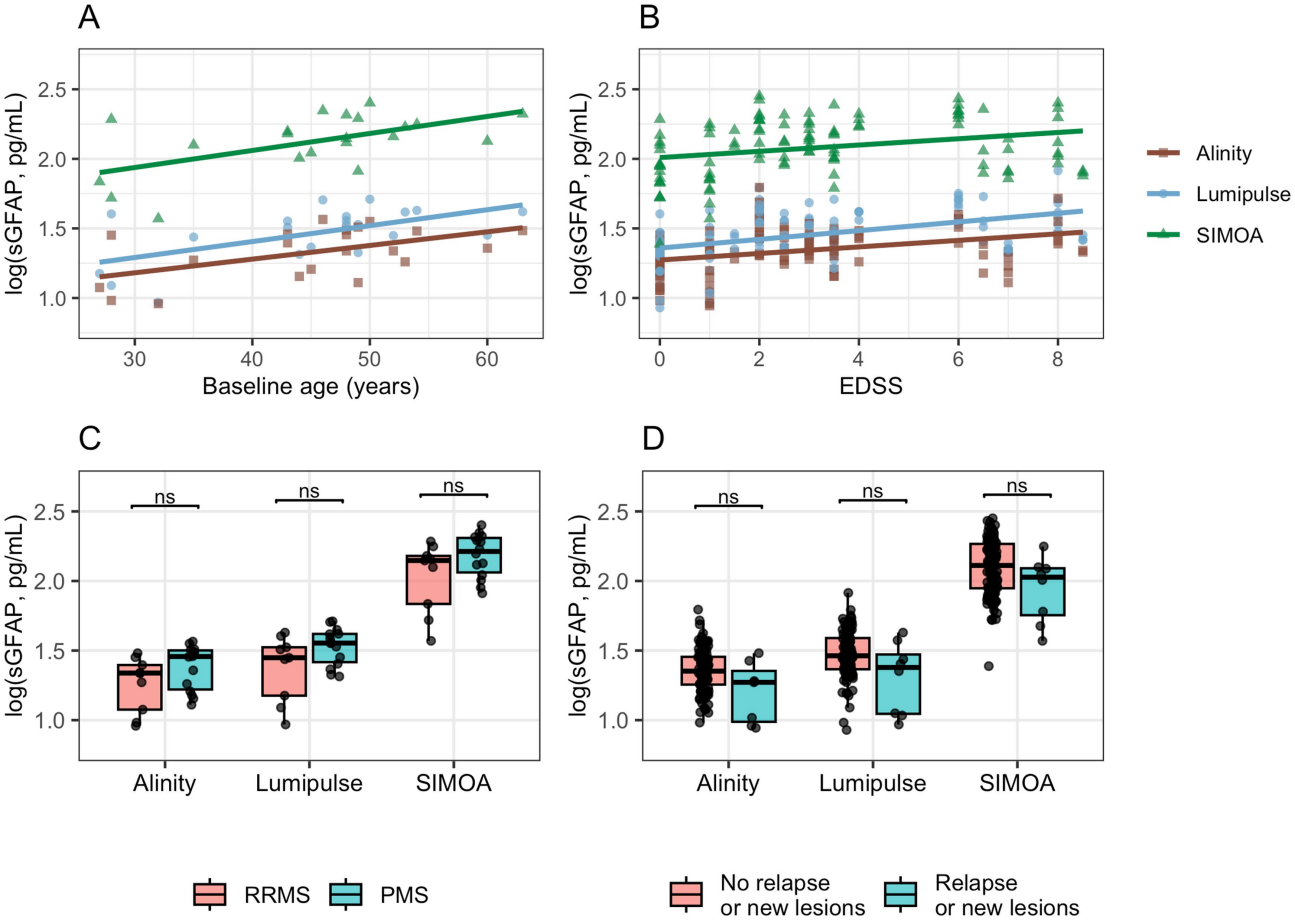
Associations of log-sGFAP with demographic and clinical variables across analytical platforms. Clinical activity was defined as the presence of a clinical relapse or an increase in MRI lesion burden at the time of blood sampling. EDSS, Expanded Disability Status Scale; PMS, Progressive MS; RRMS, Relapsing–remitting MS.

sGFAP values measured across the three platforms correlated significantly with baseline age (SIMOA: *r* = 0.57, *p* = 0.0066; Lumipulse: *r* = 0.58, *p* = 0.0056; Alinity: *r* = 0.53, *p* = 0.013; Figure 4A) and with EDSS (SIMOA: *ρ* = 0.31, *p* = 0.0009; Lumipulse: *ρ* = 0.45, *p* < 0.0001; Alinity: *ρ* = 0.37, *p* = 0.0002; Figure 4B). No significant correlations were observed between ΔEDSS and Δlog values across platforms or with disease duration. None of the platforms revealed significant differences in sGFAP levels between relapsing–remitting and progressive phenotypes (Figure 4C) or between patients with and without relapse or new MRI lesions (Figure 4D).

## Discussion

Our findings provide solid support for the clinical utility of fully automated platforms in the quantification of sGFAP. However, the analyses revealed the presence of systematic bias, comprising both constant and proportional components, which must be taken into account when interpreting results.

A constant bias was expected due to the differing dynamic ranges of SIMOA and the automated platforms: whereas SIMOA typically reports values in the hundreds, Lumipulse and Alinity yield concentrations in the tens. This discrepancy was reflected in statistically significant negative mean biases and non-zero intercepts. Of greater clinical relevance, however, was the presence of proportional bias, evidenced by a statistically significant Passing–Bablok regression slope and a positive correlation between the mean values and their corresponding differences. These findings suggest a systematic proportional deviation across the measurement range that could affect the comparability of results between platforms. Nevertheless, the magnitude of this correlation was modest—particularly for Lumipulse—and although the regression slopes differed significantly from unity, they closely approximated it, thereby mitigating the practical implications of the proportional bias. Still, its potential clinical impact in specific diagnostic or monitoring settings cannot be entirely ruled out.

The longitudinal analysis provides further evidence for the functional interchangeability of platforms. Correlations of Δlog values across sequential measurements showed a consistent linear association, even in the absence of absolute agreement. Mixed-effects models confirmed that longitudinal changes in sGFAP were comparable across platforms, underscoring their utility for patient monitoring. In these models, ΔLumipulse emerged as a significant predictor of ΔSIMOA, while ΔAlinity showed only a trend toward significance, reinforcing the analytical robustness of these associations. By contrast, no significant links were observed with clinical activity measures (ΔEDSS, relapses, new MRI lesions) or with clinical phenotype, suggesting that sGFAP dynamics primarily reflect analytical concordance rather than immediate disease activity.

This capacity to reliably capture intra-individual variation is particularly relevant in longitudinal patient monitoring, as in cases of NMOSD (11, 12), MS (27–29), Alzheimer’s disease, and other neurodegenerative conditions (30).

The systematic underestimation relative to SIMOA may be attributed to intrinsic technological differences between platforms, such as the lower analytical sensitivity of conventional immunoassays or variability in the immunorecognition of the biomarker, given the multiple isoforms and post-translational modifications of GFAP (1, 31). These limitations have been previously documented by Xu et al., who reported that both ELISA and SIMOA assays may be affected by biological matrix complexity and procedural demands (16). Further studies are needed to determine whether these differences stem from technical limitations or systematic overestimation inherent to SIMOA.

Nonetheless, in clinical terms, Lumipulse showed the most robust association with EDSS, and even Alinity outperformed SIMOA, which represents a highly positive finding. No significant discrepancies were observed between platforms in predicting short-term disease activity; however, these results should be interpreted with caution given the limited sample size and the low prevalence of clinical events. It should be noted that the primary aim of this work was the analytical comparison of platforms, and the limited number of clinical events in our cohort precludes firm conclusions regarding the monitoring value of sGFAP.

Mean sGFAP concentrations obtained using the SIMOA platform fell within the reference ranges previously established by Rodero-Romero et al. (145.8 pg./mL for individuals under 55 years of age and 280 pg./mL for those over 55) (32). For the Lumipulse platform, mean values were substantially below the upper reference limit proposed by Agnello et al. (92 pg./mL). However, it is worth noting that this threshold was derived from a cohort with a mean age of 55 years and included individuals over 70, whose average values approached 60 pg./mL (24). Within this context, our results appear consistent with prior literature, falling near the upper range reported for individuals under 50 years of age.

Regarding the Alinity platform, no reference values in healthy populations have been published to date. Nonetheless, the manufacturer has proposed a cut-off of 35 pg./mL, primarily intended for detecting axonal injury in TBI (21–23). In our cohort, sGFAP values quantified using this platform showed a pattern similar to the other two systems, generally remaining below the proposed threshold. Only 12 (13%) samples exceeded this value. Our results are consistent with the recently published study by Arslan et al., which also demonstrated a strong correlation between Alinity and SIMOA measurements in healthy controls (33).

Taken together, the observed correlation with SIMOA values, combined with the reproducibility and clinical applicability of fully automated platforms, supports their positioning as valid and efficient tools for routine clinical use. Their adoption could reduce reliance on ultrasensitive technologies, provided that platform-specific cut-offs are appropriately tailored to each clinical context. Additionally, age-related effects should be taken into account (24, 32, 34, 35).

This potential not only facilitates more efficient clinical implementation, but also promotes broader access to emerging neurological biomarkers by enabling their use in less specialized healthcare settings. Moreover, automated platforms are considerably less costly. In particular, the Abbott platform offers a further advantage, as it is currently the only one with CE marking, reinforcing its regulatory viability and clinical applicability.

These findings are especially relevant in a context where serum GFAP is gaining increasing importance and is being positioned as a key component in diagnostic and prognostic algorithms for a range of neurological disorders (3, 27).

From a clinical implementation perspective, beyond the adjustment of cut-offs, it would be highly relevant to develop inter-platform conversion models. The absolute values reported by different assays differ markedly in scale, and the systematic biases observed underscore the need for harmonization strategies. While simple linear correction factors may partially compensate for constant bias, the presence of proportional bias suggests that more flexible regression-based approaches are warranted. Polynomial or other nonlinear regression models may represent a first step, and if their performance proves insufficient, advanced machine learning approaches—such as support vector machines or deep learning—could be explored, ideally incorporating relevant covariates such as age or EDSS to improve predictive accuracy. Importantly, such algorithms could be integrated into clinical decision support systems (CDSS) (36), enabling real-time conversion within laboratory information systems. Moreover, these harmonization efforts should ideally be extended to healthy control cohorts to ensure generalizability and facilitate the establishment of unified reference ranges.

Multiple assays are currently available for measuring serum GFAP, including more recent platforms such as NULISA (37) and i-STAT; however, these have not been directly compared with SIMOA (38, 39). To our knowledge, only one recent study has evaluated more than one platform (Ella, Alinity, and Meso Scale Discovery) in parallel with SIMOA, but Lumipulse was not included (33). Our study therefore represents the first direct head-to-head comparison of SIMOA, Lumipulse, and Alinity for GFAP measurement using the same set of longitudinal samples. Another key strength of this work lies in its specific focus on the longitudinal dynamics of the biomarker and the novelty of its approach.

However, this study has several limitations. First, it focused exclusively on patients with MS, which restricts the generalizability of the findings to other neurological conditions. Second, the limited number of clinical events (EDSS worsening, relapses, or MRI activity) precluded firm conclusions regarding the utility of sGFAP for monitoring disease activity, despite the analytical agreement observed across platforms. In addition, the small number of patients limited the evaluation of its potential to discriminate between phenotypes, and the absence of healthy controls prevented the establishment of reference ranges. Finally, the moderate sample size did not allow for the development and validation of a robust inter-platform conversion model to further enhance clinical applicability. Future multicenter studies in larger cohorts are needed to address these limitations and establish the clinical utility of sGFAP on the evaluated platforms.

## Conclusion

Our findings support fully automated platforms as reliable and accessible tools for sGFAP quantification. Although they underestimate levels compared with SIMOA, recalibration of diagnostic thresholds or conversion algorithms may mitigate discrepancies. The strong correlations and consistent longitudinal dynamics endorse their use in clinical monitoring, particularly in settings requiring rapid and widely available solutions. Among them, Lumipulse showed the most robust performance, positioning it as the frontrunner for clinical implementation and paving the way to democratize sGFAP testing in neurological care.

## Data Availability

The raw data supporting the conclusions of this article will be made available by the authors, without undue reservation.

## References

[1] Gogishvili D Honey MIJ Verberk IMW Vermunt L Hol EM Teunissen CE. The GFAP proteoform puzzle: how to advance GFAP as a fluid biomarker in neurological diseases. J Neurochem. (2025) 169:e16226. doi: 10.1111/jnc.16226, PMID: 39289040 PMC11658191

[2] Janigro D Mondello S Posti JP Unden J. GFAP and S100B: what you always wanted to know and never dared to ask. Front Neurol. (2022) 13:597. doi: 10.3389/fneur.2022.835597, PMID: 35386417 PMC8977512

[3] Heimfarth L Passos FRS Monteiro BS Araújo AA d S Quintans Júnior LJ Quintans J d SS. Serum glial fibrillary acidic protein is a body fluid biomarker: a valuable prognostic for neurological disease - a systematic review. Int Immunopharmacol. (2022) 107:108624. doi: 10.1016/j.intimp.2022.108624 35255304

[4] Meier S Willemse EAJ Schaedelin S Oechtering J Lorscheider J Melie-Garcia L. Serum glial fibrillary acidic protein compared with Neurofilament light chain as a biomarker for disease progression in multiple sclerosis. JAMA Neurol. (2023) 80:287–97. doi: 10.1001/jamaneurol.2022.5250, PMID: 36745446 PMC10011932

[5] Abdelhak A Huss A Kassubek J Tumani H Otto M. Serum GFAP as a biomarker for disease severity in multiple sclerosis. Sci Rep. (2018) 8:14798. doi: 10.1038/s41598-018-33158-8, PMID: 30287870 PMC6172254

[6] Abdelhak A Antweiler K Kowarik MC Senel M Havla J Zettl UK. Serum glial fibrillary acidic protein and disability progression in progressive multiple sclerosis. Ann Clin Transl Neurol. (2024) 11:477–85. doi: 10.1002/acn3.51969, PMID: 38111972 PMC10863922

[7] Zou Y Wang Y Ma X Mu D Zhong J Ma C. CSF and blood glial fibrillary acidic protein for the diagnosis of Alzheimer’s disease: a systematic review and meta-analysis. Ageing Res Rev. (2024) 101:102485. doi: 10.1016/j.arr.2024.102485, PMID: 39236854

[8] Chatterjee P Vermunt L Gordon BA Pedrini S Boonkamp L Armstrong NJ. Plasma glial fibrillary acidic protein in autosomal dominant Alzheimer’s disease: associations with aβ-PET, neurodegeneration, and cognition. Alzheimers Dement. (2023) 19:2790–804. doi: 10.1002/alz.12879, PMID: 36576155 PMC10300233

[9] Shen XN Huang SY Cui M Zhao QH Guo Y Huang YY. Plasma glial fibrillary acidic protein in the Alzheimer disease continuum: relationship to other biomarkers, differential diagnosis, and prediction of clinical progression. Clin Chem. (2023) 69:411–21. doi: 10.1093/clinchem/hvad018, PMID: 36861369

[10] Álvarez-Sánchez L Ferré-González L Peña-Bautista C Balaguer Á Amengual JL Baquero M. New approach to specific Alzheimer’s disease diagnosis based on plasma biomarkers in a cognitive disorder cohort. Eur J Clin Investig. (2025) 55:e70034. doi: 10.1111/eci.70034, PMID: 40119567 PMC12257263

[11] Schindler P Aktas O Ringelstein M Wildemann B Jarius S Paul F. Glial fibrillary acidic protein as a biomarker in neuromyelitis optica spectrum disorder: a current review. Expert Rev Clin Immunol. (2023) 19:71–91. doi: 10.1080/1744666X.2023.2148657, PMID: 36378751

[12] Kim H Lee EJ Lim YM Kim KK. Glial fibrillary acidic protein in blood as a disease biomarker of Neuromyelitis Optica Spectrum disorders. Front Neurol. (2022) 13:865730. doi: 10.3389/fneur.2022.865730, PMID: 35370870 PMC8968934

[13] Mastrangelo A Vacchiano V Zenesini C Ruggeri E Baiardi S Cherici A. Amyloid-Beta co-pathology is a major determinant of the elevated plasma GFAP values in amyotrophic lateral sclerosis. Int J Mol Sci. (2023) 24:13976. doi: 10.3390/ijms241813976, PMID: 37762278 PMC10531493

[14] Anaya-Cubero E Fernández-Irigoyen J Santamaría E. Application of single molecule array (SIMOA) in cerebrospinal fluid. Methods Mol Biol Clifton NJ. (2025) 2914:25–39. doi: 10.1007/978-1-0716-4462-1_3 40167908

[15] Dong R Yi N Jiang D. Advances in single molecule arrays (SIMOA) for ultra-sensitive detection of biomolecules. Talanta. (2024) 270:125529. doi: 10.1016/j.talanta.2023.125529, PMID: 38091745

[16] Xu L Ramadan S Akingbade OE Zhang Y Alodan S Graham N. Detection of glial fibrillary acidic protein in patient plasma using on-Chip graphene field-effect biosensors, in comparison with ELISA and Single-molecule Array. ACS Sens. (2022) 7:253–62. doi: 10.1021/acssensors.1c02232, PMID: 34908400 PMC8805154

[17] Pilotto A Quaresima V Trasciatti C Tolassi C Bertoli D Mordenti C. Plasma p-tau217 in Alzheimer’s disease: Lumipulse and ALZpath SIMOA head-to-head comparison. Brain J Neurol. (2025) 148:408–15. doi: 10.1093/brain/awae368, PMID: 39679606 PMC11788209

[18] Dakterzada F Cipriani R López-Ortega R Arias A Riba-Llena I Ruiz-Julián M. Assessment of the correlation and diagnostic accuracy between cerebrospinal fluid and plasma Alzheimer’s disease biomarkers: a comparison of the Lumipulse and Simoa platforms. Int J Mol Sci. (2024) 25:4594. doi: 10.3390/ijms25094594, PMID: 38731812 PMC11083365

[19] Quaresima V Pilotto A Trasciatti C Tolassi C Parigi M Bertoli D. Plasma p-tau181 and amyloid markers in Alzheimer’s disease: a comparison between Lumipulse and SIMOA. Neurobiol Aging. (2024) 143:30–40. doi: 10.1016/j.neurobiolaging.2024.08.007, PMID: 39208716

[20] Gasque-Rubio R Cubas-Nuñez L Tortosa-Carreres J Forés-Toribio L Castillo-Villalba J Carratalá-Boscá S. Comparative assessment of Simoa and Lumipulse for measuring serum neurofilament light chain in multiple sclerosis patients. Acta Neurol Scand. (2024) 2024:1950913. doi: 10.1155/2024/1950913 PMC11559265 39539651

[21] Menditto VG Moretti M Babini L Mattioli A Giuliani AR Fratini M. Minor head injury in anticoagulated patients: performance of biomarkers S100B, NSE, GFAP, UCH-L1 and Alinity TBI in the detection of intracranial injury. A prospective observational study. Clin Chem Lab Med. (2024) 62:1376–82. doi: 10.1515/cclm-2023-1169, PMID: 38206121

[22] Kobeissy F Arja RD Munoz JC Shear DA Gilsdorf J Zhu J. The game changer: UCH-L1 and GFAP-based blood test as the first marketed in vitro diagnostic test for mild traumatic brain injury. Expert Rev Mol Diagn. (2024) 24:67–77. doi: 10.1080/14737159.2024.2306876, PMID: 38275158

[23] Bazarian JJ Welch RD Caudle K Jeffrey CA Chen JY Chandran R. Accuracy of a rapid glial fibrillary acidic protein/ubiquitin carboxyl-terminal hydrolase L1 test for the prediction of intracranial injuries on head computed tomography after mild traumatic brain injury. Acad Emerg Med. (2021) 28:1308–17. doi: 10.1111/acem.14366, PMID: 34358399 PMC9290667

[24] Agnello L Gambino CM Ciaccio AM Giglio RV Scazzone C Tamburello M. Establishing sex- and age-related reference intervals of serum glial fibrillary acid protein measured by the fully automated lumipulse system. Clin Chem Lab Med. (2025) 63:1402–8. doi: 10.1515/cclm-2025-0093, PMID: 40059067

[25] Thompson AJ Banwell BL Barkhof F Carroll WM Coetzee T Comi G. Diagnosis of multiple sclerosis: 2017 revisions of the McDonald criteria. Lancet Neurol. (2018) 17:162–73. doi: 10.1016/S1474-4422(17)30470-2, PMID: 29275977

[26] Müller J Sharmin S Lorscheider J Ozakbas S Karabudak R Horakova D. Standardized definition of progression independent of relapse activity (PIRA) in relapsing-remitting multiple sclerosis. JAMA Neurol. (2025) 82:614–25. doi: 10.1001/jamaneurol.2025.0495, PMID: 40227706 PMC11997854

[27] Abdelhak A Foschi M Abu-Rumeileh S Yue JK D’Anna L Huss A. Blood GFAP as an emerging biomarker in brain and spinal cord disorders. Nat Rev Neurol. (2022) 18:158–72. doi: 10.1038/s41582-021-00616-3, PMID: 35115728

[28] Park Y Kc N Paneque A Cole PD. Tau, glial fibrillary acidic protein, and Neurofilament light chain as brain protein biomarkers in cerebrospinal fluid and blood for diagnosis of neurobiological diseases. Int J Mol Sci. (2024) 25:6295. doi: 10.3390/ijms25126295, PMID: 38928000 PMC11204270

[29] Sánchez-Juan P Valeriano-Lorenzo E Ruiz-González A Pastor AB Rodrigo Lara H López-González F. Serum GFAP levels correlate with astrocyte reactivity, post-mortem brain atrophy and neurofibrillary tangles. Brain. (2024) 147:1667–79. doi: 10.1093/brain/awae035, PMID: 38634687 PMC11068326

[30] Sanchez E Coughlan GT Wilkinson T Ramirez J Mirza SS Baril AA. Association of plasma biomarkers with longitudinal atrophy and microvascular burden on MRI across neurodegenerative and cerebrovascular diseases. Neurology. (2025) 104:e213438. doi: 10.1212/WNL.0000000000213438 40063856

[31] de Reus AJEM Basak O Dykstra W van Asperen JV van Bodegraven EJ Hol EM. GFAP-isoforms in the nervous system: understanding the need for diversity. Curr Opin Cell Biol. (2024) 87:102340. doi: 10.1016/j.ceb.2024.102340, PMID: 38401182

[32] Rodero-Romero A Monreal E Sainz-Amo R García Domínguez JM Villarrubia N Veiga-González JL. Establishing Normal serum values of Neurofilament light chains and glial fibrillary acidic protein considering the effects of age and other demographic factors in healthy adults. Int J Mol Sci. (2024) 25:7808. doi: 10.3390/ijms25147808, PMID: 39063050 PMC11277397

[33] Arslan B Rembeza E Rasch S Andreasson U Blennow K Zetterberg H. Method comparison of plasma and CSF GFAP immunoassays across multiple platforms. Clin Chem Lab Med. (2025) 2025:667. doi: 10.1515/cclm-2025-0667, PMID: 40887826

[34] Mannix R Borglund E Monashefsky A Master C Corwin D Badawy M. Age-dependent differences in blood levels of glial fibrillary acidic protein but not ubiquitin Carboxy-terminal hydrolase L1 in children. Neurology. (2024) 103:e209651. doi: 10.1212/WNL.0000000000209651, PMID: 38986044 PMC11238939

[35] Vågberg M Norgren N Dring A Lindqvist T Birgander R Zetterberg H. Levels and age dependency of Neurofilament light and glial fibrillary acidic protein in healthy individuals and their relation to the brain parenchymal fraction. PLoS One. (2015) 10:e0135886. doi: 10.1371/journal.pone.0135886, PMID: 26317831 PMC4552591

[36] Choy KW Cornu P Dighe AS Georgiou A Peters L Sikaris KA. Clinical decision support in laboratory medicine. Clin Chem. (2024) 70:474–81. doi: 10.1093/clinchem/hvae002, PMID: 38300892

[37] Zeng X Sehrawat A Lafferty TK Chen Y Rawat M Kamboh MI. Novel plasma biomarkers of amyloid plaque pathology and cortical thickness: evaluation of the NULISA targeted proteomic platform in an ethnically diverse cohort. Alzheimers Dement. (2025) 21:e14535. doi: 10.1002/alz.14535, PMID: 39989429 PMC11848535

[38] Sperry JL Luther JF Okonkwo DO Vincent LE Agarwal V Cotton BA. Early GFAP and UCH-L1 point-of-care biomarker measurements for the prediction of traumatic brain injury and progression in patients with polytrauma and hemorrhagic shock. J Neurosurg. (2024) 141:917–26. doi: 10.3171/2024.1.JNS232569, PMID: 39076152 PMC11174922

[39] Korley FK Datwyler SA Jain S Sun X Beligere G Chandran R. Comparison of GFAP and UCH-L1 measurements from two prototype assays: the Abbott i-STAT and ARCHITECT assays. Neurotrauma Rep. (2021) 2:193–9. doi: 10.1089/neur.2020.0037, PMID: 33937911 PMC8086519

