# Supplementary material for: Cross-platform analytical assessment of serum GFAP quantification in multiple sclerosis: SIMOA versus two automated immunoassays
Source: Front Neurol. 2025 Oct 20;16:1682198. doi: 10.3389/fneur.2025.1682198 (PMC12580120; doi:10.3389/fneur.2025.1682198)
Supplement: Supplementary file 1 [file Data_Sheet_1.docx]

**
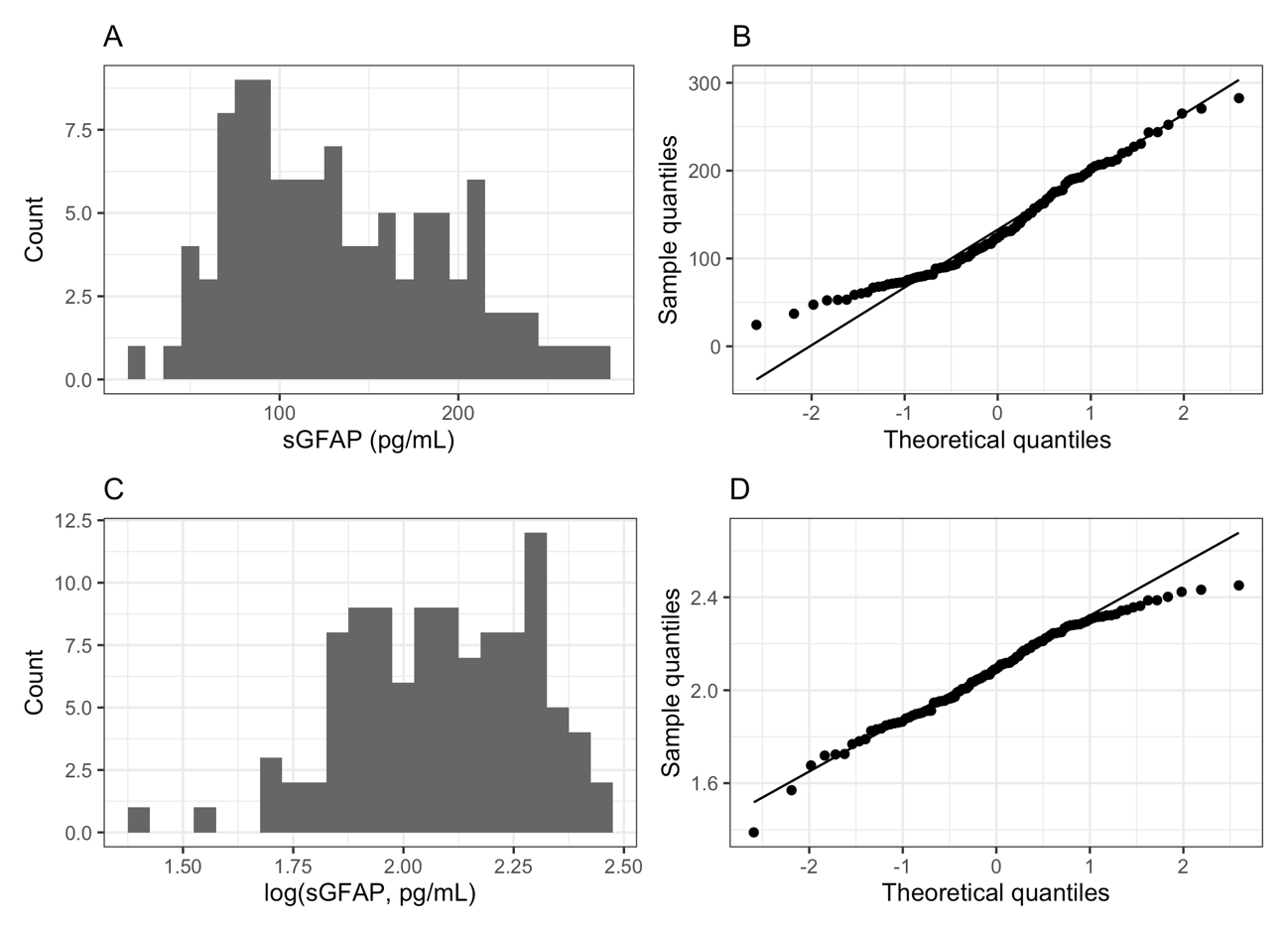
Supplementary Figure S1. Distribution of SIMOA sGFAP values before and after log-transformation.**

Panels A (histogram) and B (QQ plot) show raw concentrations (Shapiro–Wilk p = 0.005). Panels C (histogram) and D (QQ plot) show log-transformed values (p = 0.05).

**
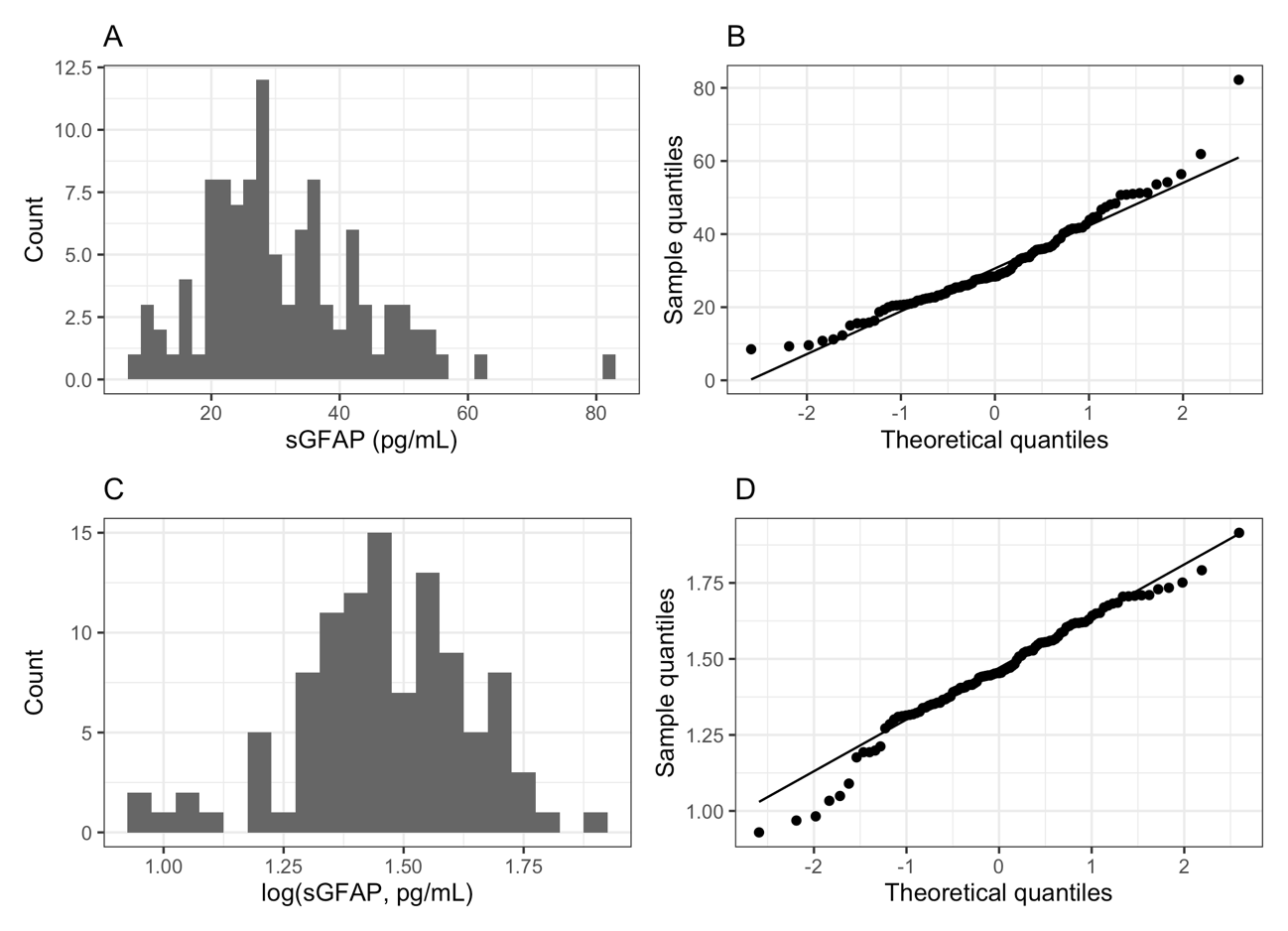
Supplementary Figure S2. Distribution of Lumipulse sGFAP values before and after log-transformation.**

Panels A (histogram) and B (QQ plot) show raw concentrations (Shapiro–Wilk p = 0.0014). Panels C (histogram) and D (QQ plot) show log-transformed values (p = 0.04).


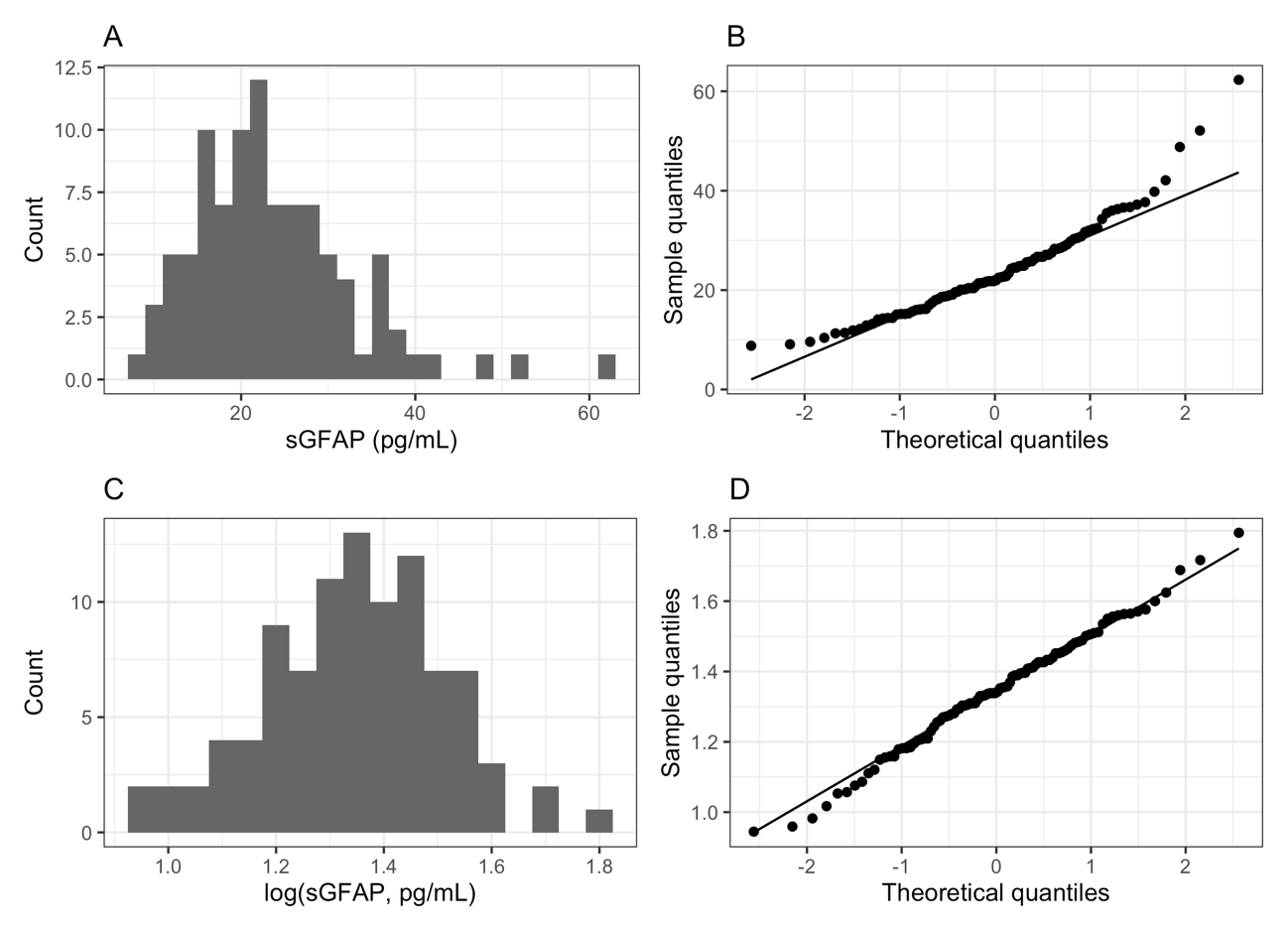
**Supplementary Figure S3. Distribution of Alinity sGFAP values before and after log-transformation.**

Panels A (histogram) and B (QQ plot) show raw concentrations (Shapiro–Wilk p = 0.0001). Panels C (histogram) and D (QQ plot) show log-transformed values (p = 0.94).

**Supplementary Table S1. Pairwise Comparison and Agreement of Raw sGFAP Measurements Across SIMOA, Lumipulse, and Alinity Platforms.**

|  | **SIMOA vs Lumipulse** | **SIMOA vs Alinity** | **Lumipulse vs Alinity** |
| --- | --- | --- | --- |
| **Intercept [95%CI]*** | 5.38 [3.69, 7.72] | 5.05 [3.33, 7.17] | 0.97 [0.54, 2.54] |
| **Slope [95%CI]*** | 0.19 [0.17, 019] | 0.13 [0.12, 0.15] | 0.72 [0.66, 0.78] |
| **sGFAP Correlation (r)** | 0.87 ( P < 0.0001) | 0.85 (P < 0.0001) | 0.86 ( P < 0.0001) |
| **Bias [95%CI]†** | 102.66 [93.26, 112.05] | 7.5 [-8.61, 23.6] | 197.82 [181.71, 213.93] |
| **LL [95%CI]†** | 109.32 [98.93, 119.71] | 8.82 [-9, 26.64] | 209.83 [192.01, 227.65] |
| **UL [95%CI] †** | 6.97 [5.79, 8.15] | -4.42 [-6.44, -2.4] | 18.36 [16.34, 20.37] |
| **Bias-Mean Correlation (r)** | 0.97 (P < 0.0001) | 0.97 (P < 0.0001) | 0.36 (P = 0.0002) |

*: Passing–Bablok regression estimates; †: Bland–Altman analysis estimates.

Pearson correlation was consistently applied to evaluate associations between platforms and the bias–mean relationship in Bland–Altman analyses, as these variables are continuous and approximately normal after logarithmic transformation. This strategy avoids alternating between Pearson and Spearman, which could create confusion in the interpretation of coefficients, and allows for a more homogeneous comparison across results. Moreover, the sample size of nearly 100 observations provides sufficient robustness for the application of parametric methods.

LL: Lower Limit of Agreement; UL: Upper Limit of Agreement; CI: Confidence Interval; sGFAP: Serum Glial Fibrillary Acidic Protein; r: Pearson correlation coefficient.

**Supplementary Figure S4. Passing–Bablok regression analysis of raw sGFAP values across analytical platforms.**

Panels A–C depict pairwise comparisons between SIMOA–Lumipulse, SIMOA–Alinity, and Lumipulse–Alinity, respectively.

**Supplementary Figure S5. Bland–Altman plots and correlation analysis of raw sGFAP values across platforms.**


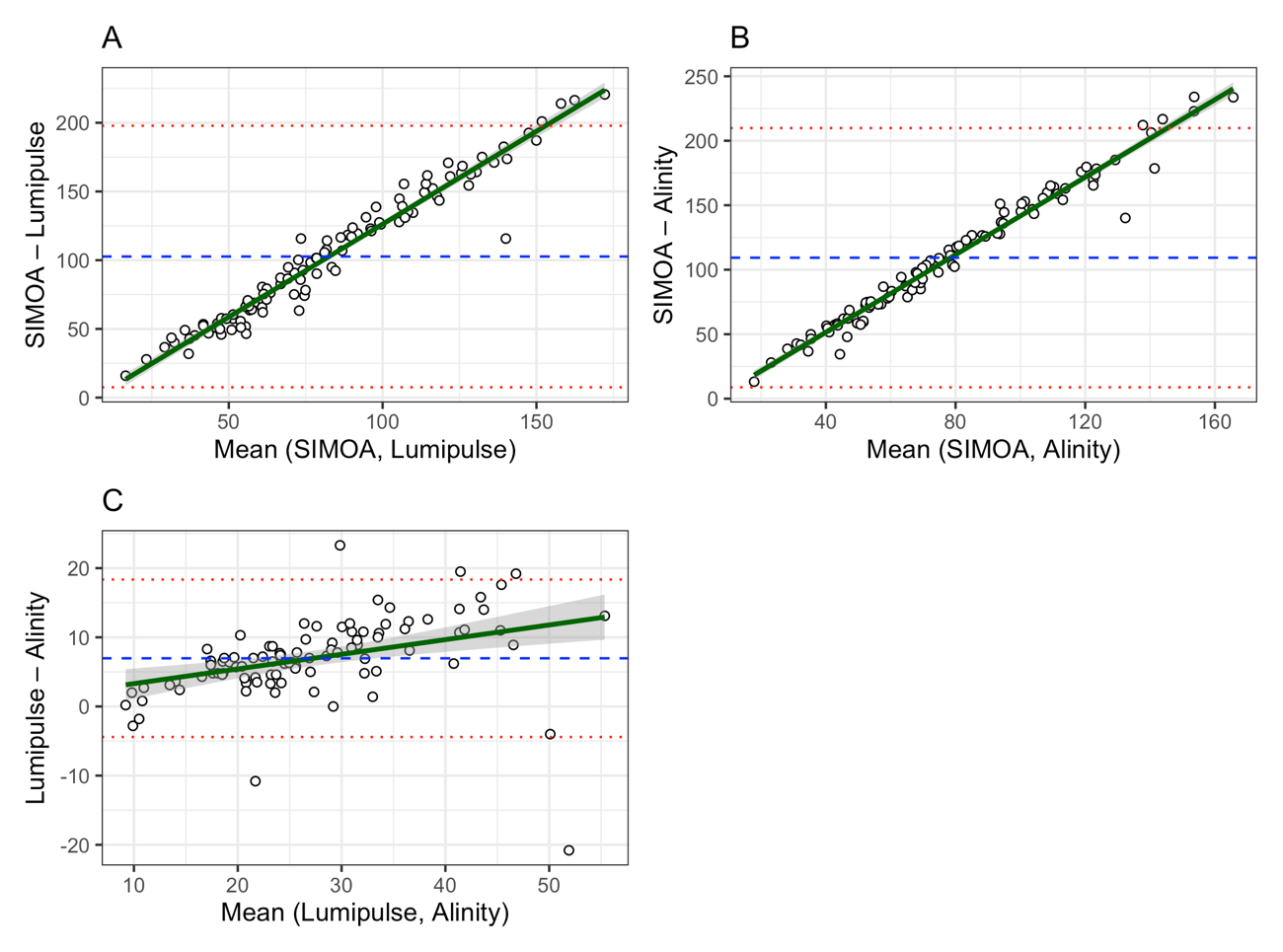


The dashed blue line indicates the mean bias, red lines denote the upper and lower limits of agreement, and the green line depicts the regression line for the correlation between means and differences.


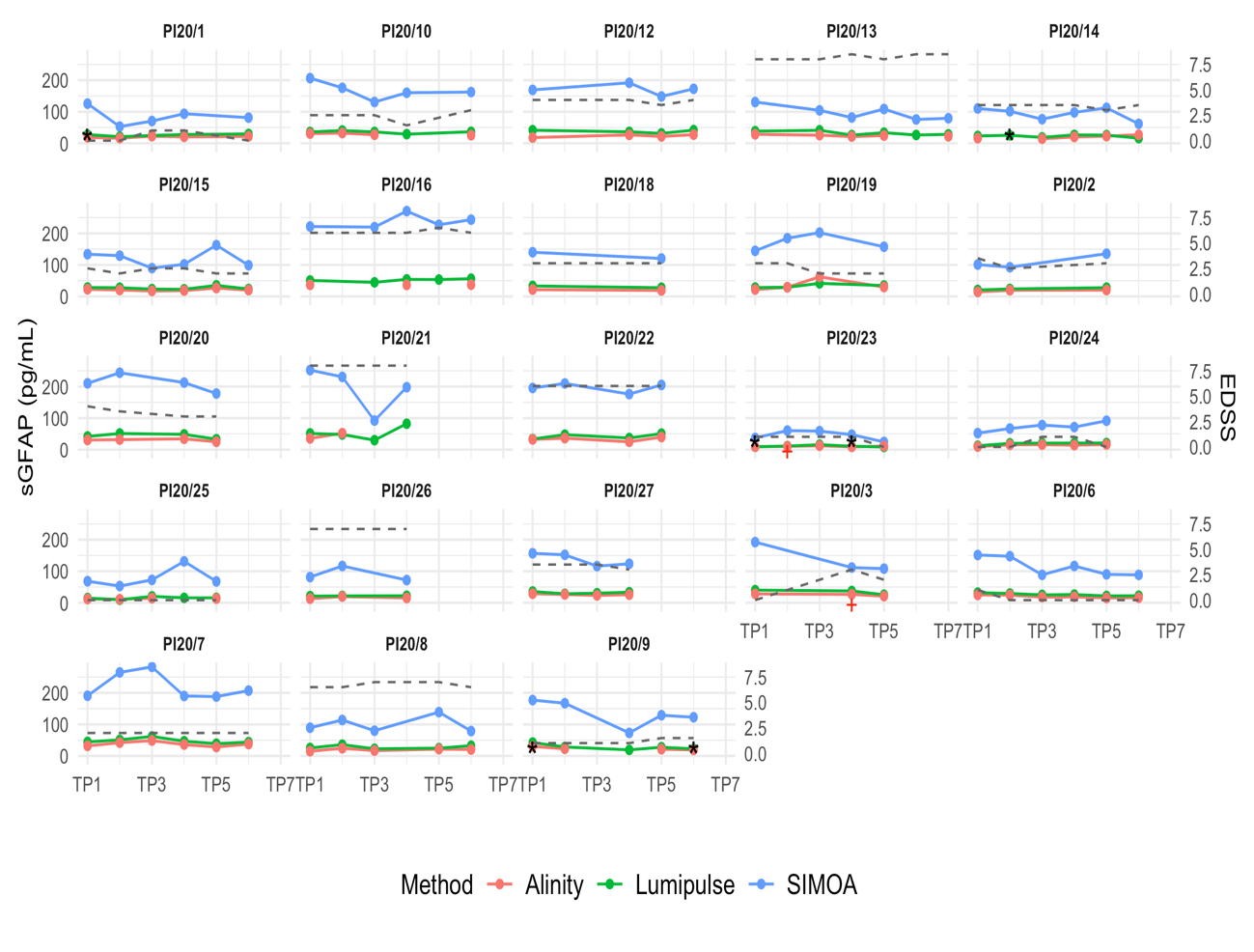
**Supplementary Figure S6. Longitudinal trajectories of raw serum sGFAP concentrations in multiple sclerosis patients over a 2-year follow-up with 4-month sampling intervals.**

Missing data points indicate unavailable serum aliquots for the respective platform. The grey dashed line represents EDSS trajectory.

TP: Time point; sGFAP: serum GFAP.

* indicates the time point at which the patient showed an increase in lesion burden during the interval since the previous visit; + indicates the time point at which the patient experienced a clinical relapse during the interval since the previous visit.
